# Medical humanities in a rapidly changing world. Is there any worth in it?

**DOI:** 10.15694/mep.2018.0000192.1

**Published:** 2018-09-04

**Authors:** Eduardo Morera Serna

**Affiliations:** 1 Hospital Universitario Son Espases

**Keywords:** Humanism, medical humanities, dataism, burn-out syndrome

## Abstract

This article was migrated. The article was marked as recommended.

Humanism has shaped mankind during the last centuries, but technology and recent social changes have shifted the paradigm towards a data driven society where humans are no longer at the center of the world. Medicine is not alien to this new situation; clinical practice is rapidly changing, and medical curricula must adapt to it. Is there any place for humanities in medical training? In this paper we analyze the present situation of humanism in a technology flooded health environment, the roll of humanities in contemporary medical curricula and we propose a series of measures to be taken with the purpose of educating the next generation of doctors.

## Perspective

Humanism is the philosophical doctrine that places the human being at the centre of the universe, the “realm of mankind”. Born during the Renaissance from the recovery of classical Roman authors such as Gellius, humanism replaced theism and heralded the end of the Middle Ages and the coming of a new society where reason prevailed over dogma, freedom over servitude and man over God.

Throughout the next centuries humanism brought enlightenment, supported empirical science, inspired human rights declaration and became the backbone of the eighteenth-century revolutions that carried humanity into the contemporary era. The idea of the progress of mankind, so popular in the nineteenth century, branched off from humanism and even religious scholars devoutly embraced it.

Modern medicine is no less in debt to humanism, and not only because of the already mentioned relationship with science, but also because of its view on the right to a good healthcare as a core component of democratic societies. Many physicians are famous not only for their scientific work but also for their dedication to music (Aleksandr Borodin, Edward Jenner, Theodor Billroth), literature (John Keats, Anton Chekov, Louis Ferdinand Celine, William Carlos William, Mikhail Bulgakov, sir Arthur Conan-Doyle), philosophy (Maimonides, John Locke, Albert Schweitzer, Friedrich Schiller) or politics (Arnulfo Arias, Juscelino Kubitschek, José Rizal, William Henry Harrison). Medicine and humanism have walked hand in hand throughout centuries.

But all this has changed. Swiftly and recently.

Humanism is no longer the prevailing ideology, neither in society nor in medicine. As Yuval Noah Harari brilliantly explained in his book Homo Deus, technological advances and economic growth have shifted society towards a data-aware profit-driven world where the human being has been kicked off the throne and replaced by data and algorithms. Dataism is the new paradigm and since big data governs our lives, what is the place for humans in this new society? Is there any point in humanism? Arts and humanities are progressively being marginalized from the academic curricula at schools and questions arise over the necessity to maintain programs on liberal arts, philosophy or classic studies at many universities due to their low demand and their lack of ability to render quantifiable outcome in terms of economic benefit, prestige or political power.

Modern medicine too is a data driven activity. Healthcare politics heavily depend on plain numbers: surgical waiting list, cost per patient and procedure, bed rotation index, number of emergencies attended, patients per doctor ratio. Physician work tends as well to be reduced to ciphers: number of cases per doctor, time in office, complication rate, patient satisfaction rate, impact factor, H index. Efficiency and sustainability are the magic words in an underfunded public health system and numbers are required. Technology will dramatically reduce the number of healthcare workers in the next decades. Big data and algorithms have provided almost flawless diagnostic software, Toyota has built robots that already deliver medication to patients, wearable devices measure medical parameters that not long ago needed a healthcare worker to take, surgical robots have de facto eliminated the need for a surgeon to be in the same place as the patient. What will the roll of doctors be in twenty years when the current medical students of medicine will be running the system? Will they just be some sort of skilled technicians? What will society require from them? How should we train them?

These are difficult questions with no clear answer at present. If humanism is witnessing its decline, should Medical Humanities be included in an already overloaded medical curricula?

The answer is probably yes but let us take some considerations into account.

1. Including the humanities of any type into medical schools, although convenient, may be too little too late. We must bear in mind that the most important measure to be taken in order to produce future humanistic physicians would be to defend the importance of humanities at primary and secondary schools. Doctors are highly qualified scientists who fight death and suffering and, as such, are highly considered by society. From this position of privilege relevant physicians should speak-up at educational forums stressing the necessity of non-scientific subjects to be taught at schools.
2. History of Medicine is at present the only non-scientific subject included in most medical curricula in Spain. It is widely viewed by students as a minor distraction from basic and clinical science and its real impact in their training and future practice is minimal; a different angle could be tried. Why not employ the time in other activities instead of attending lectures and answering multiple choice questions at the end of the term? Why not expose the students to real life situations such as visiting palliative care units or mental health wards and writing about their experience? Debates on ethical issues? Art seminars with real artists? Medicine undergraduates are intelligent and avid readers; their personal interests go beyond science. Let’s just give them time and opportunities to engage with humanities and let them decide how.
3. Science without a humanistic approach is useless and dangerous. The moment medicine is bereft of its primary goal, working for the individual and collective health, it becomes a meaningless activity. Technology and evolution of society in the last decades have greatly changed clinical practice; if physicians are not aware of this and do not stand-up against this ongoing dehumanization, the future of medicine and therefore society will be obscure. Doctors must be social leaders and teach by example; students need to meet in their practices not just skillful surgeons or encyclopedic internists, but sympathetic doctors who have a rapport with their staff and take their time to communicate with patients, who are socially aware and cultural consumers and show values and integrity. Be as you would wish them to be.
4. Medicine is a long-term career, sometimes rewarding, sometimes deceiving, not always amazing. Burn-out syndrome is an epidemic phenomenon amongst doctors and an expensive and serious problem for health systems. Humanistic education helps to cope with the ups and downs of daily clinical work building resilient physicians. Investing in medical humanities to achieve top professionals may be one of the soundest ideas of medical schools. Let´s give value to it: organize congresses and symposiums, write papers, attend meetings, exchange experiences with colleagues. Let’s put medical humanities into regular practice. We may not change the future, but we will make us fitter to it.

## Take Home Messages

• The intrinsic value of medical humanities is building more resilient and socially-aware physicians.
• Different angles should be tried to engage medical students with the humanities.
• Doctors must be social leaders and fight the progressive dehumanization of the health system.

## Notes On Contributors

Eduardo Morera Serna MD, EBE-ORL, BCFPS is a staff member at the ENT Department in Son Espases Universitary Hospital. National Delegate of the European Academy of Facial Plastic Surgery and Examiner of the European Board of Facial Plastic Surgery, holds a special interest in humanities and medical education.

## References

[ref1] Nisbet R. (1980) History of the idea of progress Routledge. New York.

[ref2] Hariri YN. (2016) Homo Deus. A brief history of tomorrow Harper Collins Publishers. London.

[ref3] Wald HS McFarland J Markovina I (2018) Medical humanities in medical education and practice. Med Teach. Aug 23:1–5. 10.1080/0142159X.2018.1497151 30134753

[ref4] Liu EY Batten JN Merrell SB Shafer A (2018) The long-term impact of a comprehensive scholarly concentration program in biomedical ethics and medical humanities. BMC Med Educ. Aug 28;18(1):204. 10.1186/s12909-018-1311-2 30153822 PMC6114241

[ref5] Chati R Huet E Grimberg L Schwarz L (2017) Factors associated With burnout among French digestive surgeons in training: results of a national survey on 328 residents and fellows. Am J Surg. 2017 Apr;213(4):754–762. 10.1016/j.amjsurg.2016.08.003 27771033

