# Supplementary material for: Medical humanities in a rapidly changing world. Is there any worth in it?
Source: MedEdPublish (2016). 2018 Sep 4;7:192. [Version 1] doi: 10.15694/mep.2018.0000192.1 (PMC10701827; doi:10.15694/mep.2018.0000192.1)
Supplement: Peer review history

# Reviewer report: Reviewer response for version 1

**DOI:** 10.21956/mep.19670.r29374

**Published:** 2018-09-19

**Authors:** Irina Markovina

**Affiliations:** 1 Sechenov First Moscow State Medical University (Sechenov University)

This review has been migrated. The reviewer awarded 5 stars out of 5 As a linguist who has worked in the field of medical education for more than 30 years, I find the article brilliant. An interested, passionate, constructive opinion piece or rather personal view. I think the article is well written stylistically and is well structured. Both its style and structure make the article very dynamic and easy to follow the author’s arguments. I am also impressed by the author’s background, especially by mentioning the Russian names of A. Borodin and M. Bulgakov, which does not happen very often in articles written in English.The concept of the content is well thought over with the starting point being the philosophical interpretation of humanism. The author moves on to the historical outline of medicine and humanism walking hand in hand throughout centuries, and finishes with a ruthless statement of the arrival of a new dataism paradigm, which reduced the medical profession to numbers, algorithms and indices.To me as a teacher, this means that medical students are now taught more to remember that to think.I completely agree with the author that for medical educators it is not enough just to observe marginalization of humanities from the academic curricula at educational institutions. Final considerations and Take Home Message sound like an Action Plan answering the question if there is anything that could be or rather should be done. To my mind, it is one of the best articles in this issue.

Reviewer Expertise:

NA

No decision status is available

# Reviewer report: Reviewer response for version 1

**DOI:** 10.21956/mep.19670.r29372

**Published:** 2018-09-05

**Authors:** Gert Van Zyl

**Affiliations:** 1 University of the Free State

This review has been migrated. The reviewer awarded 4 stars out of 5 I really enjoyed reading this paper and wants to give recognition to the importance of the topic and the role it has to play in health sciences curricula. We also need to acknowledge that some disciplines have progressed faster in implementation of the approach in teaching practices. Enjoyed the list of considerations. The Spain experience might not be relevant to all but still illustrates the concept well.

Reviewer Expertise:

NA

No decision status is available

# Reviewer report: Reviewer response for version 1

**DOI:** 10.21956/mep.19670.r29370

**Published:** 2018-09-04

**Authors:** P Ravi Shankar

**Affiliations:** 1 American International Medical University

This review has been migrated. The reviewer awarded 4 stars out of 5 The author addresses some of the important issues facing current and future medical practice. Medical practice will be increasingly dominated by technology and big data. Algorithms and artificial intelligence are making steady inroads into medical practice. The author rightly asks what will be the role of the future doctor. How will they interact with technology? Modern medicine is being increasingly driven by commercial interests and efficiency. In our medical humanities module we introduce the theme that being an empathetic physician requires time and effort and faces challenges from the modern system where time is money and lesser the amount of time spend with a patient, the greater may be the profit. The point about the humanities reducing burnout among doctors may be an important one to favor its introduction and expansion in the medical curriculum. Burnout is expensive and any measures to reduce it will be looked on favorably. The evidence base linking the medical humanities with reducing burnout should be strengthened through research.

Reviewer Expertise:

NA

No decision status is available

# Reviewer report: Reviewer response for version 1

**DOI:** 10.21956/mep.19670.r29373

**Published:** 2018-09-04

**Authors:** Stijntje Dijk

**Affiliations:** 1 Erasmus Medical Center

This review has been migrated. The reviewer awarded 3 stars out of 5 An interesting opinion piece by a physician with a passionate call for the return of humanities not only in medical education but in primary and secondary schools as well. I couldn't agree more with this articles statement about the crucial role of role models within medicine. Knowledge in humanities can be lectured, but some skills and values cannot be delivered through word and text only - let alone be assessed in students. I wonder whether the author would see any additional role for 1) adjustments in the methods/standards for selecting students entering medical school, and 2) for assessing them before leaving it.I would, however, still like to "vouch" for the economists referred to in this paper. Although the area of health economics is much data-driven, I would argue that the core principle of the field does, in fact, has much in common with "ours". "Economics are concerned with the efficient allocation of scarce resources in society and the equity implications of such allocation. Equity and efficiency issues cannot be solved independently (Arrow. 1963)." But perhaps the solution would remain the same as the author proposes: with a more humane approach perhaps we in health would learn to understand the positive intentions behind our economics colleagues to help as many people as possible with the scarce goods that exist, and they would learn to understand the humane side of a physician's work that seeing, talking with, understanding a patient and dealing with the administrative burdens around them within a 5 minute time frame can take the care out of health care.One hope there might be for the grim future predicted in this paper is what one might find in the Human Development Report 2015: Work for Human Development. Although it predicts there are many jobs likely to be replaced by automation (telemarketers, order clerks, mathematical technicians, data entry keyers and others), many health jobs such as physicians, psychologists, dentists and oral and maxillofacial surgeons fall within the top 20 jobs least likely to be replaced. Additionally, reports such as the US Bureau of Labor Statistics Data (Turner et al 2013) do predict still predict jobs in health care to continue to be the fastest growing occupations in the upcoming year. What exact role they will play? I don't know. But as this author rightly proposes, humanities is one key difference between our work and those jobs replaceable by automatics. That means to me, this call to the integration of the humanities will be more important than ever, or our of data-driven activity will have driven us away from what makes medicine so special. Doctors must be social leaders - absolutely. They have a role to play in raising such issues as the author has raised to those around them: educators, politicians, economists, and colleagues that have an opportunity to lead by example.

Reviewer Expertise:

NA

No decision status is available

# Reviewer report: Reviewer response for version 1

**DOI:** 10.21956/mep.19670.r29371

**Published:** 2018-09-04

**Authors:** Trevor Gibbs

**Affiliations:** 1 AMEE

This review has been migrated. The reviewer awarded 4 stars out of 5 A very disheartening but passionate paper regarding the placing of humanities squarely within out healthcare curricula. The author makes some excellent points and neatly draws our attention to those well know authors, musicians etc that were also doctors. As I read that part of the papers I was also thinking if in fact they were also good doctors? - did their interest in the arts make them a good doctor. The well known UK neurologist - Johnathan Miller turned to the arts from his early days as a medical student- was he a better doctor because of that? I particularly like the 4 paragraph summary at the end of this paper. Perhaps an added section may focus around how the Humanities can bring some sanity into this crazy evolving world. A very neat, personal paper that I would encourage others to read

Reviewer Expertise:

NA

No decision status is available
